# “Progressive myoclonic ataxia and developmental/epileptic encephalopathy associated with a novel homozygous mutation in *TCN*2 gene”

**DOI:** 10.1002/mgg3.2282

**Published:** 2023-10-06

**Authors:** Mohammed Ahmed Mohammed Oshi, Jaber Alfaifi, Youssef Ali M. Alqahtani, Mohammed Fahad Aljabri, Naglaa M. Kamal, Jwaher Althopaity, Khalid A. Althobaiti, Abdullah M. Almalki, Salma A. S. Abosabie, Sara A. Abosabie, Hanan Sakr Sherbiny, Saif K. Almanjoomi, Enas A. A. Abdallah

**Affiliations:** ^1^ Neurology Division Gaafar Ibnauf Children's Emergency Hospital Khartoum Sudan; ^2^ Department of Child Health, College of Medicine University of Bisha Bisha Saudi Arabia; ^3^ Department of Child Health, College of Medicine King Khalid University Abha Kingdom of Saudi Arabia; ^4^ Alhada Armed Forces Hospital Taif Kingdom of Saudi Arabia; ^5^ Department of Pediatrics and Pediatric Hepatology, Kasr Alainy Faculty of Medicine Cairo University Cairo Egypt; ^6^ Department of Medical Genetics King Fahad Medical City Riyadh Saudi Arabia; ^7^ Faculty of Medicine Julius‐Maximilians‐Universität Würzburg Wurzburg Bavaria Germany; ^8^ Faculty of Medicine Charité Universitätsmedizin Berlin Berlin Germany; ^9^ Department of pediatrics, Faculty of Medicine Zagazig University Zagazig Egypt

**Keywords:** ataxia, exome sequencing, myoclonic seizures, neurodevelopmental regression, *TCN* 2, Transcobalamin II defect, tremor

## Abstract

**Background:**

Transcobalamin II (*TCN*2) defect is a rare metabolic disorder associated with a range of neurological manifestations, including mild developmental delay, severe intellectual disability, ataxia, and, in some cases, seizures. Cobalamin, an essential nutrient, plays a crucial role in central nervous system myelination.

**Clinical Presentation:**

We present a family with an index patient who exhibited progressive neurodevelopmental regression starting at 9 months of age, accompanied by myoclonic seizures, ataxia, and tremor. No significant hematological abnormalities were observed. Exome sequencing analysis identified a novel homozygous mutation, c.3G>A – P(Met1I), affecting the acceptor site of intron 4 of the *TCN*2 gene (chromosome 22: 31003321, NM_000355.4), leading to likely pathogenic variant potentially affecting translation. Following treatment with hydroxocobalamin, the patient demonstrated partial clinical improvement. He has a sibling with overt hematological abnormalities and subtle neurological abnormalities who is homozygous to the same mutation. Both parents are heterozygous for the same mutation.

**Conclusions:**

In infants presenting with unexplained non‐specific neurological symptoms, irrespective of classical signs of vitamin B12 deficiency, evaluation for *TCN*2 defect should be considered. Early diagnosis and appropriate management can lead to favorable outcomes.

## Graphical abstract

Transcobalamin defect should be evaluated in infants with unexplained non‐specific neurological problems, whether or not there is classic evidence of vitamin B 12 deficiency.

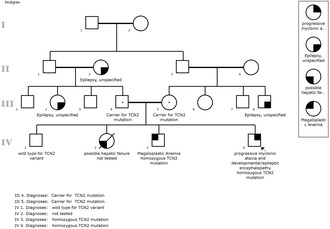

## 1 INTRODUCTION

Cobalamin, also known as vitamin B12 (Cbl), plays a crucial role in cell metabolism and DNA synthesis (Trakadis et al., 2014). Transcobalamin deficiency can present with complex diagnostic and therapeutic challenges due to its varied clinical manifestations. Transcobalamin II (*TCN*2) is a plasma protein that binds to cobalamin, facilitating cellular uptake through receptor‐mediated endocytosis. Alongside intrinsic factor (IF) and haptocorrin (HC), *TCN* plays a vital role in the absorption and transport of vitamin B12 (Guéant et al., 2014; Huemer & Baumgartner, 2019; Kapadia, 1995; Trakadis et al., 2014).

*TCN*2 mutations can lead to *TCN*2 deficiency or defect, resulting in intracellular cobalamin depletion and a rare autosomal recessive multisystem disorder. Clinical presentations of *TCN*2‐related disorders include pancytopenia, megaloblastic anemia, failure to thrive, diarrhea, psychomotor regression, and, in rare cases, epilepsy (Kapadia, 1995). The initial symptoms typically manifest in infancy, with an average onset between 2 and 4 months of age (Guéant et al., 2014). Transcobalamin deficiency, a rare autosomal recessive inborn error of cobalamin transport, clinically manifests in early infancy with a prevalence of 1 in 1,000,000 (Hall, 1992). Cobalamin is essential for the initiation and maintenance of myelination in the central nervous system, and untreated *TCN* deficiency can result in delayed development and regression in approximately 30% of affected individuals (Hall, 1992).

In addition to intellectual delay, transcobalamin can cause ataxia, pyramidal deficits in the limbs, microcephaly, drug‐resistant epilepsy, choreiform movements, hypotonia, and drug‐resistant epilepsy (Hall, 1992). In adulthood, megaloblastic anemia and subacute combined spinal cord degeneration may also occur (Thomas et al., 1982).

Teplitsky et al. reported chronic clinical manifestations of *TCN*2 deficiency, including learning difficulties, low intelligence, vertigo, clonus, and personality disorders. Interestingly, affected children and young adults may have normal or slightly lower serum vitamin B12 levels despite not being anemic (Teplitsky et al., 2003). Infants with *TCN*2 defect may present with megaloblastic anemia, feeding difficulties, developmental delay, microcephaly, failure to thrive, hypotonia, lethargy, irritability, involuntary movements, focal or multifocal seizures, and cerebral atrophy (Benbir et al., 2007).

Cobalamin deficiencies have been associated with various neurological symptoms, including irritability and neurodevelopmental regression. Pathophysiological mechanisms may involve defects in myelination, demyelination, axonal degeneration, neurotoxic cytokine imbalances, and neuronal lactate accumulation (Benbir et al., 2007; Obeid et al., 2006). Initial magnetic resonance imaging (MRI) may reveal hypomyelination and brain atrophy, but subsequent MRI scans show improvement with B12 supplementation (Dror & Allen, 2008; Nashabat et al., 2017). Severe truncal hypotonia, neurologically significant developmental delay, and hypogammaglobulinemia have also been linked to *TCN*2 deficiency (Black, 2008; Lehotsky et al., 2015). Rigaudiere et al. documented neuro‐ophthalmological consequences of *TCN*2 mutations, including subclinical maculopathy and retinopathy (Kose et al., 2020; Martino et al., 2021; Rigaudiere et al., 2022).

Progressive myoclonic encephalopathy (PME) is a group of rare, genetically heterogeneous diseases characterized by progressive neurocognitive impairment, cortical myoclonus, and various types of epileptic seizures. Unlike epileptic encephalopathies that typically present with polymorphic seizures in early infancy, PMEs usually manifest in late childhood or adolescence (Genton et al., 2016).

In this report, we describe an infant with progressive neurological features in whom next‐generation sequencing facilitated the identification of a homozygous novel mutation in the *TCN*2 gene. To the best of our knowledge, this is the first research describing this novel mutation in the *TCN*2 gene.

## 2 CLINICAL PRESENTATION

A 14‐month‐old male infant from the southern part of Saudi Arabia was born at full term following an uneventful 38‐week pregnancy. His birth parameters were within normal ranges, with a weight of 2970 grams and a head circumference of 33.7 cm. There was an absence of prenatal complications such as polyhydramnios, or reduced fetal movements, as well as any immediate postnatal complications. Newborn screening for common metabolic disorders, including urea cycle defects, organic acidemias, aminoacidopathies, and medium‐chain acyl‐CoA dehydrogenase (MCAD) deficiency, using tandem mass spectrometry, showed no abnormalities.

At 9 months of age, the child's development took an alarming turn. Previously progressing typically, he began experiencing a gradual regression of motor skills and vocalization abilities. This regression was coupled with early signs of oromotor dysfunction. Notably, the child never reached independent sitting or walking milestones. By 11 months, he displayed unsteadiness, intention tremors, frequent head nodding episodes occurred throughout the day and eventually becoming continuous, and myoclonus primarily affecting the upper extremities which progressed, along with abnormal rhythmic eye movements. These clinical features correlated with abnormal electroencephalogram (EEG) findings, solidifying the progressive nature of his condition.

**FIGURE 1:**
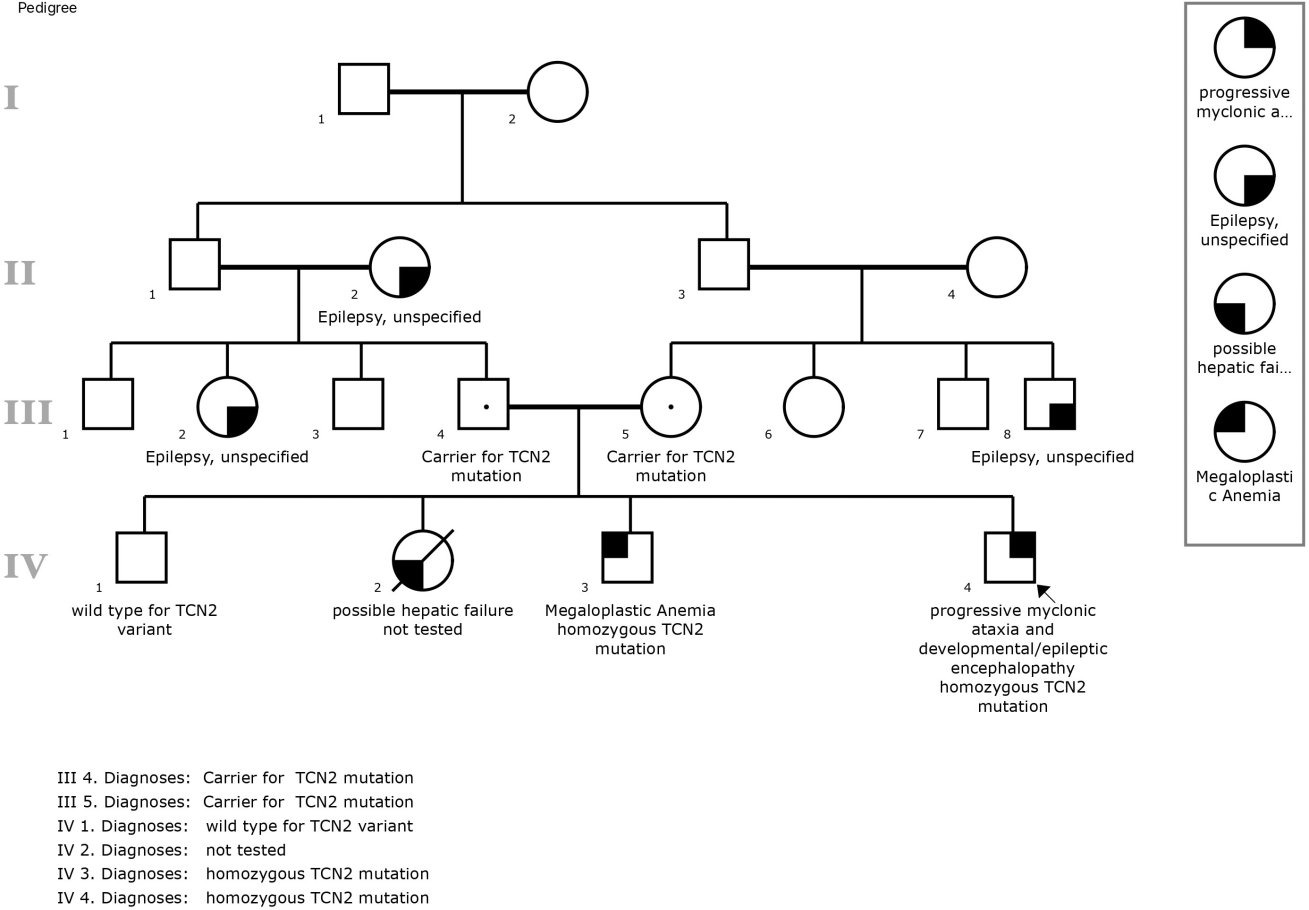
Extended family pedigree.

The patient was born to first cousins couples, he had an older sister who succumbed to unexplained hepatic failure at 7 years old and an older brother with anemia of unknown origin. Importantly, the family history also indicated two generations with generalized childhood/adolescent‐onset epilepsies of unknown cause. The family pedigree is shown in Figure 1. His vaccination history remained up‐to‐date.

At 12 months, clinical examination revealed a spectrum of symptoms including irritability, poor visual attention, nystagmoid eye movements, startle response, tongue fasciculations, bulbar dysfunction, myoclonus affecting upper limbs, generalized hypotonia/ hyporeflexia, truncal unsteadiness, polyminimyoclonus, and mild hepatomegaly (2 cm in the right mid clavicular line). While head circumference remained within normal limits, and no signs of cyanosis, pallor, or neurocutaneous markers were noticed. Dilated fundal examination yielded normal results. Partial alleviation of myoclonus was achieved with the use of levetiracetam, clonazepam, and multivitamins.

Initial laboratory investigations, including blood counts, liver and kidney function tests, electrolyte levels, bone biochemistry (magnesium, phosphorus, and calcium), ammonia, and lactate yielded normal results. However, at 10 months, an elevated mean corpuscular volume was observed, accompanied by slightly elevated transaminases. Vitamin B12 metabolism abnormalities were suspected due to the persistent elevation of mean corpuscular volume despite normal hemoglobin level. Subsequent analysis revealed normal serum vitamin B12 (351 pg/mL, range: 200–860). However, the serum homocysteine level was elevated at 40 μmol/L (reference range: 5.5–17), and the urinary methylmalonic acid level was elevated 5.6 μmol/mmol crt (reference range: 0.4–2.5 μicromoles/mmol creatinine), indicating impaired B12 metabolism.

Interictal electroencephalography (EEG) demonstrated polyspikes and slow waves at 4 to 6 Hz on multiple occasions, coupled with photosensitivity. Addition of clobazam led to improvements in background EEG activity and epileptiform discharges.

His brain MRI performed at 9 months of age showed no detectable abnormalities. However, due to frequent worsening episodes of myoclonus, and progressive weakness, the patient's condition prompted a repeat MRI at 14 months, revealing cerebral atrophy and poor myelination.

### 2.1 Genetic testing and definitive diagnosis

With the progressive nature of the symptoms and the complex clinical picture, molecular genetic testing via exome sequencing was pursued.

#### 2.1.1 Molecular genetic testing: Exome sequencing

##### Methods

Genomic DNA was fragmented, and the exons of known genes in the human genome, along with the corresponding exon–intron boundaries, were enriched using Roche KAPA capture technology (KAPA HyperExome Library). The enriched DNA was amplified and sequenced simultaneously using illumina technology (next‐generation sequencing, NGS) on an illumina system (novo gene). The target regions were sequenced with an average coverage of 125‐fold, ensuring a 15‐fold coverage for 100% of the regions of interest and a 20‐fold coverage for approximately 99.9% of the regions. According to ACMG recommendations, the pathogenic mutations were classified into five classes: Class 1—Pathogenic, Class 2—Likely pathogenic, Class 3—Variant of uncertain significance (VUS), Class 4—Likely benign, and Class 5—Benign.

NGS data were aligned to the hg19 genome assembly. Variant calling and annotation were performed using in‐house developed bioinformatics pipelines. Identified variants were filtered against external and internal databases, focusing on rare variants with a minor allele frequency (MAF) in gnomAD of 1% or less, and known artifacts and variants in highly homologous regions were removed. The classification of variants followed ACMG guidelines, taking into account database entries (including HGMD), bioinformatics prediction tools, and literature status. It should be noted that changes in pathogenicity classification over time cannot be excluded. Variants annotated as common polymorphisms in databases or literature or those classified as (likely) benign were not considered.

Putatively pathogenic differences between the patient's sequence and the wild‐type sequence (human reference genome according to UCSC Genome Browser, hg19, GRCh37) mentioned and interpreted in this report were evaluated using an established in‐house quality score. Variants that did not meet the quality threshold were verified using polymerase chain reaction amplification followed by conventional Sanger sequencing. Sample identity was ensured through internal quality management procedures.

##### Results

Exome sequencing analysis identified a homozygous variant, c.3G>A – P(Met1I), affecting the acceptor site of intron 4 of the *TCN*2 gene (chromosome 22: 31003321, NM_000355.4), (OMIM: *613441), leading to likely pathogenic variant. This variant causes the loss of the start codon, potentially affecting translation.

To the best of our knowledge, this variant has not been previously described in the literature (HGMD 2022.1). Its frequency in the general population has not been documented (gnomAD v2.1.1 controls). Based on these findings, the variant is classified as likely pathogenic and is causally related to the observed clinical phenotype.

### 2.2 Treatment and follow‐up

The therapeutic regimen was initiated with a commencement dose of 1 mg of intramuscular hydroxocobalamin daily over a week, subsequently transitioning to a twice‐weekly schedule. The clinical and biochemical response was diligently tracked through the surveillance of complete blood picture, serum lactate dehydrogenase, and plasma homocysteine levels. Clinical neurological examination and serial electroencephalography were used as additional monitoring measures for epileptic encephalopathy (Table 1).

**TABLE 1:** Effect of treatment on patient's clinical and biochemical manifestations.

Assessment	Pre‐treatment	Post‐treatment for 18 months
Clinical manifestation		
Cognitive dysfunction	Present	Improved (can respond to commands)
Myoclonic epilepsy	Present	Partial improvement (decreased to 2–3 episodes/day)
Movement disorder (ataxia & tremors)	Present	Partial improvement with the ability == to sit without support
Laboratory investigations		
Pancytopenia	Absent	N/A
Serum vitamin B12 level	Normal	Normal
Serum homocysteine level	Elevated	Normalized
Urinary methylmalonic acid level	Elevated	Normalized

*Note*: N/A indicates not applicable, as the patient did not exhibit pancytopenia.

Myoclonus episodes decreased to two to three episodes, he can sit without support, and responds to commands.

The patient demonstrated a positive but partial response to cobalamin therapy, marked by heightened energy levels, improved oral feeding, and gradual achievement of motor milestones. Swift decline in plasma homocysteine levels was noted, paralleled by enhanced decrease and normalization of mean corpuscular volume values evident in successive complete blood count analyses.

Presently, at 17 months of age, the patient continues to grapple with moderate to severe speech delay and poor social interaction. However, there has been slow improvement in motor development and the frequency of myoclonus has decreased **(**Table 1 **)**.

### 2.3 Family screening and genetic counseling

Following the identification of the index case, a meticulous familial screening strategy was executed. Molecular genetic testing, tailored to the identified variant, was extended to encompass both parents and the two surviving siblings. The assessment unveiled that both parents carried the identical mutation in a heterozygous state. The sibling afflicted with anemia was disclosed to harbor the same mutation in a homozygous pattern, warranting the initiation of high‐dose vitamin B12 supplementation. Conversely, the remaining sibling demonstrated genetic normalcy (Figure 1). It is of note that testing of previously affected family members is not possible and you cannot conclude if the conditions are related.

Incorporating a comprehensive approach, the family was subjected to informed genetic counseling sessions elucidating the nuances of the disease and its hereditary implications. Parents were equipped with the essential knowledge to comprehend the inheritance pattern and the potential risks for future offspring. An ongoing dialogue was established to facilitate informed decision‐making, supported by a collaborative engagement with a geneticist for sustained guidance and surveillance.

## 3 DISCUSSION

We present an extended family with a novel *TCN*2 mutation causing complex neurological manifestations, including progressive cognitive dysfunction, myoclonic epilepsy, and movement disorder – ataxia and tremor. As far as we are aware, this is the first research reporting this *TCN*2 mutation with usual neurological manifestations without significant hematological consequences.

Comparisons with previous studies reveal variations in clinical findings. Trakadis et al. reported that 87.5% of *TCN*2 patients have hematological findings, including pancytopenia, along with delayed milestones, hypotonia, dyslexia, decreased IQ, vertigo, plantar clonus, and personality disorder. However, our index case predominantly exhibited neurological manifestations, and seizures were not reported (Genton et al., 2016). Schiff et al. (2010) found that gastrointestinal and hematological complications occur at an earlier age in *TCN*2 patients, while neurological complications appear later. Their identified mutation affected the interaction between transcobalamin and transcobalamin receptors, which differs from the mutation found in our patient. Our patient exhibited a different mutation site and mechanism resulting in a loss of the start codon, potentially affecting protein formation by mRNA. These differences in clinical manifestations may be related to the types of variants and sites, as well as epigenetic factors.

Aggressive treatment, such as parenteral or intramuscular high‐dose (1 mg) injection on a weekly basis, is highly recommended (Thomas & Hoffbrand, 1997). Hydroxycobalamin treatment has shown better clinical results compared to cyanocobalamin. Successful clinical outcomes were also reported in two patients with 1 mg i.m. weekly methylcobalamin). Folic acid and betaine administrations have also been reported in TC deficiency. In our index patient, partial clinical improvement was observed after intensive treatment, and neurological examination findings were slowly improved during the short follow‐up period. More reports and prospective clinical trials will be helpful in determining the most appropriate treatment approach for TC deficiency (Schiff et al., 2010).

A case reported by Thomas et al. demonstrated similarities to our index patient, with early‐onset frequent seizures, microcephaly, hypotonia, severely retarded intellectual development, ataxia, and pyramidal deficits in the limbs. This emphasizes the importance of early diagnosis and treatment (Thomas & Hoffbrand, 1997).

No genotype–phenotype correlation has been reported in TC deficiency. Previously, insertions, deletions, splice‐site, and non‐sense mutations have been reported. In our patient, we identified a homozygous variant resulting in the loss of the start codon, which could have an effect on translation. More reports of novel variations may help evaluate the genotype–phenotype relationship better (Yildirim et al., 2017). The neurological complications of TC2 include a wide range of clinical features, such as hypotonia, psychomotor retardation or regression, seizures, movement disorders, and failure to thrive in infants. This highlights the need for a high level of suspicion in cases where neurological features are non‐specific (Yildirim et al., 2017).

Vitamin B12 deficiency can result in various neurological manifestations, including peripheral neuropathy, subacute combined spinal cord degeneration, dementia, optic atrophy, psychosis, and mood disturbances. Additionally, cerebellar ataxia, cranial nerve abnormalities, Parkinsonian syndrome, and movement disorders have been described. In our index patient, the prominent features were ataxia, truncal hypotonia, and progressive myoclonic seizures (Yildirim et al., 2017).

The first case of B12 deficiency and developmental and epileptic encephalopathy, consistent with West syndrome, was reported by Chong et al. The cases improved with B12 therapy, including the cessation of spasms and resolution of hypsarrhythmia (Chong et al., 2019). Additionally, it is important to consider the possibility of other non‐related mutations in the family history. In our index patient, there was a significant family history of generalized tonic–clonic seizure and myoclonic childhood/late adolescent onset, which may contribute to variable expression and non‐related mutations (Chong et al., 2019).

Early initiation of treatment is crucial for achieving optimal outcomes. Neurological and hematological deterioration have been reported in patients who discontinued treatment; thus, lifelong treatment is required for the prevention of these complications (Honzik et al., 2010). There is no clear consensus about the dosage, dose intervals, route of administration (i.m., oral), and the form of cobalamin for the management of TC deficiency.

## 4 CONCLUSION

This research highlights the challenges in diagnosing and managing complex developmental disorders within consanguineous families. The evolving clinical symptoms, abnormal EEG findings, and puzzling homocysteine‐methylmalonic acid dynamics exemplify the intricacies of the described index case.

Effective management demands a multifaceted approach and vigilant monitoring. The positive clinical response to hydroxocobalamin therapy, combined with genetic insights from family screening, underscores the importance of personalized care and genetic counseling for optimal outcomes.

Moving forward, addressing TC2‐related neurological complications calls for additional clinical data and interventional studies. It is important to note that retrospective analysis is hindered by the unavailability of tests for previously affected family members.

## AUTHOR CONTRIBUTIONS

All Authors contributed significantly to the conception and design, acquisition of data or analysis and interpretation of data; drafting the article or revising it critically for important intellectual content; final approval of the version published.

## FUNDING INFORMATION

None.

## CONFLICT OF INTEREST STATEMENT

The authors declare that they have no competing interests.

## DECLARATIONS

We declare that the work on which this research is based is my original work (except where acknowledgments indicate otherwise) and that neither the whole work nor any part of it has been, is being, or is to be submitted for another degree in this or any other journals.

## ETHICS APPROVAL AND CONSENT TO PARTICIPATE

This study was approved by Human Research Ethics Committee, Alhada Armed Forces Hospital, Taif, Saudi Arabia. Written informed consent for publication was obtained from parents for the inclusion of their family in the current study.

## CONSENT FOR PUBLICATION

Written informed parental consent was obtained for the publication of this research.

## Data Availability

The data and materials generated and/or analyzed during the current study are available in the current manuscript.
